# The barriers to smoking cessation in Swiss methadone and buprenorphine-maintained patients

**DOI:** 10.1186/1477-7517-5-10

**Published:** 2008-03-18

**Authors:** Victoria Wapf, Michael Schaub, Beat Klaeusler, Lukas Boesch, Rudolf Stohler, Dominique Eich

**Affiliations:** 1 Psychiatric University Hospital Zurich, Research Group on Substance Use Disorders, Selnaustrasse 9, 8002 Zurich, Switzerland

## Abstract

**Background:**

Smoking rates in methadone-maintained patients are almost three times higher than in the general population and remain elevated and stable. Due to the various negative health effects of smoking, nicotine dependence contributes to the high mortality in this patient group. The purpose of the current study was to investigate Swiss methadone and buprenorphine-maintained patients' willingness to stop smoking and to clarify further smoking cessation procedures.

**Methods:**

Substance abuse history, nicotine dependence, and readiness to stop smoking were assessed in a sample of 103 opiate-dependent patients in the metropolitan area of Zurich, Switzerland. Patients were asked to document their smoking patterns and readiness to quit.

**Results:**

Only a small number of patients were willing to quit smoking cigarettes (10.7%) and, even though bupropione or nicotine replacement therapy was included in the fixed daily treatment care, only one patient received nicotine replacement therapy for smoking cessation. A diagnosis of depression in patients' clinical records was associated with readiness to stop smoking. No significant associations were found between readiness to quit smoking and age, methadone treatment characteristics, and presence of co-dependencies.

**Conclusion:**

The current prescription level of best medicine for nicotine dependence in Swiss methadone and buprenorphine-maintained patients is far from adequate. Possible explanations and treatment-relevant implications are discussed.

## Background

Growing public awareness of the public health issues of cigarette smoking has led to the implementation of smoking prevention programs, age limits for tobacco sales, and smoking bans in public spaces in many western Europe countries. These measures have brought about substantial improvements, with overall smoking rates among adults declining to 20–40% in various countries [1,2]. In Switzerland rates vary between 30 and 40% [3]. However, smoking rates for patients with a substance use disorder remain high and stable [4]. Numerous studies suggest that smoking rates are almost three times higher in opiate-dependent persons in methadone treatment programs as compared to the general population [5-7].

For unknown reasons, the majority of patients receiving methadone maintenance treatment are cigarette smokers. Due to the various negative health effects of smoking, nicotine dependence contributes to the high mortality in this patient group. Nevertheless, many psychiatrists and other mental health professionals are often reluctant to address the problem of nicotine abuse in their patients suffering from substance use disorders. Olsen et al. [8] reported that, although addiction counseling is required in methadone programs, nicotine dependence rarely receives attention.

The reluctance of care providers has been partially attributed to a fear that the stress of smoking cessation would lead to a relapse into the abuse of other substances [9]. Despite preliminary evidence that smoking cessation counseling can be provided without necessarily leading to a relapse with other substances [10], some therapists believe that smoking serves as an effective coping tool to deal with cravings for other substances such as heroin and cocaine [11]. There is some support for an association between cigarette smoking and methadone dose in that methadone patients who exhibited higher smoking rates are significantly more likely to report problems of not feeling "held" by their methadone dose and to experience a higher level of anxiety [12]. More adequate methadone dosing would probably reduce such effects.

On the other hand, the therapists' reluctance may reflect limited motivation on the patients' part. Hayaki et al. [13] demonstrated that many smokers underestimate their personal susceptibility to the negative health effects brought about by smoking (e.g., increased risk for oncological and cardiovascular disease). As stated by Kolly et al. [14], a number of patients and treatment professionals believe that smoking is a minor issue compared to illegal drug consumption. Other studies, however, have demonstrated that many patients are interested in quitting smoking [7,15-17] and that smoking cessation does not jeopardize progresses made in treatment [18]. As delineated by Baran-Furga et al. [19], initiation of methadone maintenance treatment can also be associated with positive changes in smoking behavior. But on the other hand, studies on smoking cessation programs in methadone maintenance treatment have not been very promising [20] and the large majority of these patients in smoking cessation programs have been reported to relapse at follow-up even when nicotine replacement therapy has been combined with otherwise efficacious therapy approaches such as relapse prevention or contingency management [21].

One frequently applied concept when investigating whether individuals are likely to stop smoking is the stages of change paradigm [22]. To our knowledge, there are currently only two publications, both from the U. S., considering readiness to quit smoking in methadone-maintained patients. A study by Shadel et al. [23] of smokers enrolled in a smoking cessation research protocol revealed that, among various factors (demographics, methadone dose, numbers of smoking quits, age of first regular smoking, mood and depression), only the number of cigarettes smoked per day and high scores on smoking expectancies were associated with motivation to quit smoking as assessed by a 10-point readiness to change scale. The first study [24] investigating readiness to stop smoking with the Smoking Stages of Change Algorithm [25] in methadone-maintained patients found that prior use of smoking cessation pharmacotherapy and lower methadone doses were associated with being in the preparation stage (patients reporting an intention to stop smoking within the following 30 days). The proportion of methadone patients being in different stages of change in this study [24] was similar to that observed in the general population [26].

The purpose of the current study was therefore to investigate (1) Swiss methadone and buprenorphine-maintained patients' willingness to stop smoking cigarettes, (2) to investigate their previous pharmacotherapy as regards smoking cessation, and (3) to determine whether these factors are associated with demographics, co-dependencies, methadone substitution doses and duration, and co-occurring mental health diagnoses.

## Methods

This was a cross-sectional study designed to compare patients' willingness to stop smoking cigarettes and possible associations with demographic variables as well as co-morbid and co-dependence characteristics.

### Study subjects

The study sample was recruited from all opiate-dependent outpatients in methadone or buprenorphine maintenance therapy at the specialized outpatient facility of the Psychiatric University Clinic in Zurich, Switzerland (n = 233). Bupropione or nicotine replacement therapy was included in the fixed daily treatment care and available for the physicians and the maintenance personnel along with the other pharmacological inventory in the medicine cupboard behind the methadone maintenance counter. A physician offered the opportunity to participate prior to or after a consultation (and/or receiving their regular methadone/buprenorphine dose). Of those approached, 105 patients participated in the study and two patients were excluded according to the studies' exclusion criteria (acute cocaine, amphetamine, heroin, cannabis, alcohol, sedatives and/or hallucinogen intoxication or acute psychosis) at the time of recruitment. Finally, 103 patients fully completed the study questionnaire.

Participants were guaranteed that all information would be handled confidentially and they were informed of their right to withdraw from the study at any time without any negative consequences regarding their treatment. In particular, patients were reassured that their access to medical care would not be affected in any way by their choice to participate or not. By signing the consent form, patients stated their understanding of the study procedure and their willingness to participate. Shortly afterwards, patients could complete the study questionnaire anonymously and independently in the waiting room and were paid Euro 2.50 (CHF 5) for their inconvenience. The study protocol was approved by the ethics committee at the University of Zurich and by the established community based ethics committee.

### Measures

Willingness to stop smoking cigarettes was assessed using the Stages of Change Algorithm [25]. Although the stages of change concept has been criticized [27], there is a wide consensus that people who state that they are willing to stop smoking are more likely to actually quit than those who do not, and that evidence-based smoking cessation treatments are substantially more promising for motivated smokers than for unmotivated ones [28,29]. According to the Stages of Change Algorithm, smokers who seriously considered stopping within the next six months were classed as being in the "contemplation" stage, those who did not consider quitting were defined as "pre-contemplators". Patients who intended to stop smoking within the following 30 days were considered to be in the "preparation" stage (provided that they had undergone more than one previous attempt to quit smoking). Those who did not report such attempts, but intended to stop within the next month, were also considered "contemplators". Not smoking for less than six months and not smoking for more than six-months was graded as stage of action or maintenance, respectively. The Fagerstrom Test of Nicotine Dependence FTND [30], a widely used paper-and-pencil test, was used to measure the severity of nicotine dependence. Nicotine dependence was categorized as follows: FTND scores from 0 to 2: low dependence, 3 to 5: moderate dependence, 6 to 7: high dependence, and 8 to 10: very high dependence. Furthermore, patients were asked to imagine whom they would approach (six possible answers) if they wished to reduce or stop their cigarette consumption. Data on patients' demographics, mental health and other diagnoses, substance dependencies and previous nicotine replacement and/or bupropion therapy were obtained from their medical records.

### Data management and analyses

Data were recorded using a relational database. All survey results were coded and recorded anonymously. Data were analyzed with the statistical software package SPSS, version 11.

To explore associations between readiness to stop smoking and the above mentioned variables, non-parametric tests (Kruskal-Wallis Chi-Square) were chosen, due to the skewed nature of the values' distribution. To adjust for effects of potential confounders, a mixed general linear/logistic regression model was applied. P values < 0.05 were considered statistically significant. Power calculation revealed that a sample of 100 ± 5 subjects would be needed to test each variable with a power of >60%.

## Results

### Sample characteristics

Males comprised 75% of the sample. Patients' age ranged between 18 and 50 with a mean of 33.8 (± 7.4) years. The majority was treated with methadone (74.8% in fluid form, 10.7% in form of suppositories, 1.0% in form of pills), and the remaining patients received buprenorphine (13.6%). The mean number of enrollments in maintenance treatment (including the current one) was 3.1 (± 4.8) with a mean duration of 60.0 (± 42.7) months. The mean number of opiate withdrawal attempts was 3.9 (± 3.0).

### Stages of change

The majority of respondents (71.9%) were in the pre-contemplation stage. There were 17.5% in the contemplation and 2.9% in the preparation stage (see table 1). Only a small group of study participants was in the maintenance or action stage (3.9% each). To reduce effects of skewness and to facilitate the use of statistical tests, the values of Stages of Change variables were dichotomized post hoc as follows: patients in pre-contemplation and contemplation stages were compared with those in preparation, action and maintenance stages.

**Table 1:** Sample characteristics and smoking variables in opiate-dependent patients in maintenance treatment (n = 103)

	n	%	mean; SD
Male	78	76	
Female	25	24	
Age			33.8; 7.4
			
Maintenance Substances			
Treated with buprenorphine	14	13.6	
Treated with methadone, out of them:	89	86	
- in fluid form	77	86.5	
- in form of suppositoria	11	12.4	
- in form of tablettes	1	1.1	
			
Stages of change			
- precontemplation stage	74	71.9	
- contemplation stage	18	17.5	
- preparation stage	3	2.9	
- maintenance stage	4	3.9	
- action stage	4	3.9	
			
Smoking Variables			
Current smokers		93	
Former smokers		10	
Never smokers	-	-	-
Number of cigarettes smoked per day, pcs			
- 0–10	16	15.5	
- 11–20	36	35	
- 21–30	38	37	
- >30	10	10	
FTND score			
- 0–2 (low)		17	
- 3–5 (moderate)		28	
- 6–7 (strong)		40	
- 8–10 (very strong).		15	

In a series of exact Kruskal-Wallis Chi-Square tests, a significant positive association with readiness to stop smoking was found with female gender (not ready: 21.7%, ready: 45.5%; Chi-Square = 4.369, df = 1, p < 0.05) and with the presence of depression (not ready: 30.4%, ready: 63.7%; Chi-Square = 5.783, df = 1, p < 0.05). The logistic regression confirmed the association with depression (OR = 5.78, 95 CI = 1.32–25.29, p < 0.05) but not with female gender (OR = 1.9, 95% CI = 0.47–7.92, n.s.).

No differences were found between the preparation-action-maintenance group and the pre-contemplation/contemplation group (see table 2) regarding mean age (not ready: 33.9 (± 7.6), ready: 32.5 (± 5.9); Chi-Square = 0.190, df = 1, n.s.) and the number of participants who reported co-dependence of cannabis (not ready: 21.7%, ready: 18.2%; Chi-Square = 0.855, df = 1, n.s.), cocaine (not ready: 38.0%, ready: 45.5%; Chi-Square = 0.458, df = 1, n.s.), sedatives (not ready: 39.1%, ready: 18.2%; Chi-Square = 1.637, df = 1, n.s.) or alcohol (not ready: 26.1%, ready: 18.2%; Chi-Square = 1.395, df = 1, n.s.). Likewise, there were no significant differences between those two groups regarding methadone dose (not ready: 125.4 (± 84.0), ready: 77.1 (± 34.0); Chi-Square = 1.964, df = 1, n.s.), age of first regular use of heroin (not ready: 19.7 (± 6.0), ready: 21.4 (± 7.0); Chi-Square = 0.260, df = 1, n.s.), and history of substitution therapy (number of previous substitutions (not ready: 3.1 (± 5.0), ready: 2.8 (± 1.1); Chi-Square = 0.816, df = 1, n.s.) and total duration of substitution (not ready: 59.3 (± 41.5), ready: 71.0 (± 58.3); Chi-Square = 0.131, df = 1, n.s.)). Furthermore, the mean number of opiate withdrawal attempts did not differ significantly between groups (not ready: 3.7 (± 2.6), ready: 5.7 (± 4.5); Chi-Square = 1.141, df = 1, n.s.).

**Table 2:** Kruskal-Wallis Chi-Squares for the dichotomized stages of change groups indicating readiness to stop cigarette smoking

	Not ready	Ready	Chi-Square
Number of patients	92	11	
% female	21.7	45.5	4.369*
Age	33.9; 7.6	32.5; 5.9	0.190
			
**Cigarette Smoking**			
Age of smoking onset	14.2; 3.7	14.6; 2.7	2.419
Years of smoking	19.8; 7.4	17.8; 6.4	0.585
Number of cigarettes/day	15.5; 8.1	16.0; 7.0	0.008
FTND score	5.3; 2.1	6.5; 2.0	2.171
% nicotine replacement	1.1	0.0	-
			
**Opiate and Maintenance History**			
Age at heroin onset	19.7; 6.0	21.4; 7.0	0.260
Number of opiate substitution enrollments	3.1; 5.0	2.8; 1.1	0.816
Total months of opiate substitution treatment	59.3; 41.5	71.0; 58.3	0.131
% substituted with methadone	84.8	72.8	
Current methadone dose	125.4; 84.0	77.1; 34.0	1.964
% ever had an opiate withdrawal attempt	70.6	72.7	0.282
Total number of opiate withdrawal attempts	3.7; 2.6	5.7; 4.5	1.141
			
**Co-dependence**			
% alcohol dependence	26.1	18.2	1.395
% cannabis dependence	21.7	18.2	0.855
% sedative dependence	39.1	18.2	1.637
% cocaine dependence	38.0	45.5	0.458
Number of co-dependencies other than nicotine	1.3; 0.9	1.0; 0.5	0.922
			
**Dual Diagnoses**			
% depression	30.4	63.7	5.783*
% adult ADHD	3.3	0.0	-
% schizophreniform disorder	4.3	9.1	-

* p < 0.05

Thirty-six percent of the patients stated that they would approach their case-manager (who was a psychologist, a physician, a social worker, or a nurse) if they wanted to reduce or stop their cigarette consumption, 19.5% declared that they would try to reduce smoking on their own, 13.5% did not know who they would contact, 11.0% would contact a physician from the clinic, 8.5% a specialized facility outside the clinic, and 13.8% would try to get help from various other sources.

### Nicotine dependence

The average duration of cigarette smoking was 19.6 (± 7.3) years. Almost all respondents were current smokers, with a mean FTND score of 5.3 (± 2.1) which reflects moderate dependence. Seventeen percent of subjects were classed as having a low, 30.1% a moderate, 40.8% a strong, and 15.5% a very strong level of dependence. Only 9.7% of participants were former smokers (see table 1). Even though never having smoked was not an exclusion criterion, there were no participants in this sample who had never smoked. Only one patient had received nicotine replacement. Bupropion had never been prescribed to any patient prior to the study assessment.

### Measures of co-dependence

Four out of five patients (78.6%) suffered from co-dependencies (other than nicotine and opiates) with only 2.9% of participants reporting a sole opiate dependence. Nearly every fifth respondent (18.4%) had one co-dependence, and every third individual had three and more co-dependencies (37.9%).

Cocaine was the third most commonly used drug after opiates and tobacco (62%), followed by cannabis (36.9%), alcohol (19.4%), sedatives (17.5%), and hallucinogens (1.0%).

### Dual diagnoses

Adult ADHD was diagnosed in 3.9% of subjects and 36.9% met diagnostic criteria for depression (1.9% organic depressive disorder; 4.9% cocaine-induced depression; 1.9% schizoaffective disorder; 4.9% depressive disorder, current mild depressive episode; 4.9% moderate depressive symptoms; 12.6% recurrent depressive episodes; 0.9% cyclothymia; 1.9% dysthymia; 2.9% anxiety and depressive reaction, mixed). A schizophreniform disorder was found in 4.9% of all patients.

## Discussion

Overall, the investigated sample reflected the demographic attributes of the total patient population. There were three times more males than females, which is consistent with previously reported gender compositions for similar populations in Europe [31] and in the United States [32].

## Smoking variables

Frequency of smoking was also consistent with the known rates in comparable populations [15,23]. In general, the opiate-dependent outpatients in the current study had smoked a large number of cigarettes over many years. Their nicotine dependence was substantial (as many as 57% of patients were scored as having strong or very strong dependence in the FTND-test). Most study subjects (73%) were not willing to stop smoking. This distribution is similar to other European samples in the general population of smokers, for instance, to the results of Etter et al. [33] in Geneva. However, the results differ from those in American surveys where these distributions were typically 40% (stage of precontemplation), 40% (contemplation), and 20% (preparation) in the general population of smokers [25,26,34] and 43% (contemplation) and 22% (preparation stage) in methadone-maintained patients [24]. One obvious explanation for the difference between the distribution in the study by Nahvi et al. [24] is that virtually none of our patients were ever previously treated with prescription medication for smoking cessation. By contrast, half of the patients in the study by Nahvi et al. [24] were previously treated this way. Other possible explanations might be that the physicians in Nahvi et al's [24] study worried more about possible consequences of smoking tobacco in their patients or had different treatment relevant beliefs than the physicians in our study. Such explanations could be investigated in further studies. Moreover, it is unclear if the patients in the Nahvi et al. [24] study were more concerned about the impact of their cigarette smoking and were therefore more motivated to quit smoking. It needs to be clarified whether patients on steady methadone doses truly care less than the general population about possible health consequences and if this could represent one reason for their reduced motivation to quit.

### Co-dependence and comorbidity

As observed by a number of authors, nicotine dependence can be influenced by comorbid conditions. For instance, active alcohol abusers are reported to be 60% less likely to stop smoking than alcohol abstainers [35], and depressed nicotine and alcohol dependent patients are reported to be less likely to quit smoking than non-depressed patients [9]. Nevertheless, there were no significant associations found in the present sample between alcohol dependence and willingness to stop smoking.

Among all factors examined, a significant association was only found with a diagnosis of depression. This result is consistent with other studies that have found a significant association between depression and readiness to stop smoking in general psychiatric samples [36]. Another study, however, found no such relationship in psychiatric patients [37]. Since rates of lifetime affective disorders are high in opiate-dependent populations (e.g. Nunes [38]: 16–75%), it is important to screen patients as they may show an increased willingness for smoking cessation and therefore be open to intervention opportunities.

### Study limitations

The study design was cross-sectional and correlational and may therefore suffer from several limitations and caveats common in this type of research. These include possible sampling biases and effects of confounding variables that were unaccounted for. Moreover, recall biases concerning the dependence and treatment histories may also have affected results of self-reported treatment duration and frequency. Last but not least, the generalizability of our findings in Zurich to populations in other regions and countries remains unclear.

## Conclusion

Willingness to cease smoking was only marginally prevalent in this representative sample of Swiss methadone and buprenorphine-maintained patients. With so much focus on the reduction of illicit drug use, relatively little attention has been given to nicotine addiction in this population. Therefore, it is important to investigate why there exists such a widespread complacency in patients but also in physicians and other treatment personnel. Therefore, we suggest that health professionals be required to actively offer their patients more pharmacologically-based smoking cessation treatments to facilitate quitting and to alleviate possible adverse effects that often occurring during smoking cessation. Most patients stated that they would approach their direct case-managers if they were contemplating quitting smoking and thus, case-managers may pose the most relevant contact persons who could propose a smoking cessation attempt. The development of more adequate and tailored motivation-enhancing, psycho-social and/or psychotherapeutic interventions for nicotine dependent patients in maintenance treatment could clarify whether current interventions are specific enough and if there is greater potential for smoking cessation than that which is currently achieved in these patients.
